# The challenges of investigating antimicrobial resistance in Vietnam - what benefits does a One Health approach offer the animal and human health sectors?

**DOI:** 10.1186/s12889-020-8319-3

**Published:** 2020-02-11

**Authors:** Marisa E. V. Mitchell, Robyn Alders, Fred Unger, Hung Nguyen-Viet, Trang Thi Huyen Le, Jenny-Ann Toribio

**Affiliations:** 1 International Livestock Research Institute (ILRI), 298 Kim Ma, Ba Dinh, Hanoi, Vietnam; 2 Faculty of Arts and Social Sciences, University of Sydney, Sydney, Australia; 3 Marie Bashir Institute for Infectious Diseases and Biosecurity, University of Sydney, Sydney, Australia; 4 Centre on Global Health Security, Chatham House, Royal Institute of International Affairs, London, UK; 5 Development Policy Centre, Australian National University, Canberra, ACT Australia; 6 Kyeema Foundation, Brisbane, Australia; 7 Department of Infectious Disease and Global Health, Cummings School of Veterinary Medicine, Tufts University, North Grafton, USA; 8 Center for Public Health and Ecosystem Research, Hanoi University of Public Health, Hanoi, Vietnam; 9 School of Veterinary Science, Faculty of Science, University of Sydney, Sydney, Australia

**Keywords:** Antibiotic resistance, Antimicrobial resistance, One health, Qualitative research, Pork value chain

## Abstract

**Background:**

The One Health concept promotes the enhancement of human, animal and ecosystem health through multi-sectorial governance support and policies to combat health security threats. In Vietnam, antimicrobial resistance (AMR) in animal and human health settings poses a significant threat, but one that could be minimised by adopting a One Health approach to AMR surveillance. To advance understanding of the willingness and abilities of the human and animal health sectors to undertake investigations of AMR with a One Health approach, we explored the perceptions and experiences of those tasked with investigating AMR in Vietnam, and the benefits a multi-sectorial approach offers.

**Methods:**

This study used qualitative methodology to provide key informants’ perspectives from the animal and human health sectors. Two scenarios of food-borne AMR bacteria found within the pork value chain were used as case studies to investigate challenges and opportunities for improving collaboration across different stakeholders and to understand benefits offered by a One Health approach surveillance system. Fifteen semi-structured interviews with 11 participants from the animal and six from the human health sectors at the central level in Hanoi and the provincial level in Thai Nguyen were conducted.

**Results:**

Eight themes emerged from the transcripts of the interviews. From the participants perspectives on the benefits of a One Health approach: (1) Communication and multi-sectorial collaboration; (2) Building comprehensive knowledge; (3) Improving likelihood of success. Five themes emerged from participants views of the challenges to investigate AMR: (4) Diagnostic capacity; (5) Availability and access to antibiotics (6) Tracing ability within the Vietnamese food chain; (7) Personal benefits and (8) Managing the system.

**Conclusion:**

The findings of this study suggest that there is potential to strengthen multi-sectorial collaboration between the animal and human health sectors by building upon existing informal networks. Based on these results, we recommend an inclusive approach to multi-sectorial communication supported by government network activities to facilitate partnerships and create cross-disciplinary awareness and participation. The themes relating to diagnostic capacity show that both sectors are facing challenges to undertake investigations in AMR. Our results indicate that the need to strengthen the animal health sector is more pronounced.

## Background

Antimicrobial resistance (AMR) threatens to destabilise progression in human health and animal health by reducing the ability to treat diseases and causing complications to medical procedures. Activities impacting on the environment and actions in the human health care sector and the animal health sector are all considered to be contributing to the development of pathogen resistance to antimicrobials [1–5]. Contamination of the environment with pharmaceutical waste, for example, is one of the means by which resistant genes can transfer among pathogens in the environment [4, 6]. Documented key drivers of resistance in human health settings are the use, overuse, misuse and irrational use of antimicrobials, particularly self-diagnosis and medication, over-prescription by medical practitioners, easy and/or illegal access to antimicrobial medication without a prescription and inadequate hygiene practices of health-care workers [3, 7–9]. Within the animal agriculture sector, the routine use of antibiotics for growth promotion, prophylactic and therapeutic purposes in animal production systems can lead to the emergence of resistant bacteria on farm [3, 10–12]. The use of antibiotics on farm may pose a risk to human health as food-producing animals and the farm environment can act as reservoirs of resistant bacteria [13–16]. Therefore, to address the challenges of AMR, a One Health approach, whereby the connections between the human, animal and environmental sectors are considered is required [17]. A One Health approach can build connections and communication channels across sectors to collaborate on research and development activities and the implementation of programs, policies and legislation [18].

Vietnam is a potential hot spot for the emergence of AMR due to the high burden of infectious diseases that are directly transmissible and that are foodborne, coupled with limited enforcement of regulations to penalise non-compliance, and the relatively unregulated access to antimicrobials for humans and high antimicrobial usage for livestock [1, 8, 19, 20]. Vietnam was one of the first countries to develop a National Action Plan to combat AMR in the World Health Organization (WHO) Western Pacific Region [21]. Focusing primarily on the human health sector, the National Action Plan to combat drug resistance for 2013–2020 addresses raising awareness of AMR within the community, improving the surveillance system, safeguarding access to antimicrobials, encouraging the safe use of drugs within the human health sector and the animal health sector, and supporting infection control measures [21]. To concentrate on the needs of the animal health sector, the Ministry of Agriculture and Rural Development (MARD) subsequently developed the National Action Plan to combat AMR in Livestock and Aquaculture 2017–2020 to reduce the risk of AMR through the control of antibiotic use in animal husbandry and aquaculture in Vietnam [22]. Collaboration between the animal and human health sectors, as outlined in the Ministry of Agriculture and Rural Development National Action Plan is an essential component of evidence-based policy and guidelines aimed to control antibiotic use within the human health and animal health sectors and to provide information on the spread of bacterial strains and genetic determinants of resistance [23]. Multi-sectorial participation in the development of priority setting is thought to be most successful when trust, transparency, equal representation and consensus are present among all relevant sectors [24]. Within Vietnam, it is reported that key actors have found it difficult to understand the objectives of each sectors’ AMR surveillance system, leading to a lack of mutual understanding of the shared benefits of consistent collaboration [25]. Insufficient comparable data and variation in data quality on AMR between the human health and animal health sectors poses a challenge to identify and accurately monitor resistance. A One Health surveillance system built on a harmonized approach to laboratory techniques and data management can enhance surveillance efforts [26].

To progress effective implementation of strategies to combat antibiotic resistance, it is vital to understand the perceptions and experiences of the people tasked with investigating AMR under the National Action Plans. Moreover, it is relevant to explore how One Health and government policies to address AMR are perceived by the actors charged with implementing these policies. For the purposes of this study, we specifically focused on antibiotic resistance which is a key component of AMR. The objectives of this project were to [1]: Identify potential advantages of cross-sectoral collaborations between key informants from the animal health and human health sectors to address AMR in Vietnam; and [2] Identify the potential challenges that key informants in the animal health and human health sectors face during investigation of antibiotic resistance within the pork value chain in Vietnam.

## Methods

### Study sites and participants

The semi-structured interviews were conducted in Vietnam’s capital city, Hanoi, between August and October 2018 and in Thai Nguyen Province during February 2019. The recruitment sites were chosen to compare the challenges and activities in a central and a provincial setting and explore actors’ perceptions of a One Health approach within the different levels of government, universities and international agencies. Each key informant interviewed was chosen on the basis of the following selection criteria: a current role in the identified sector and work related to antimicrobial or antibiotic resistance or antibiotic residue project/s; involvement and knowledge of antibiotic resistance in Vietnam and the animal sourced food value chains; working within the International Livestock Research Institute, SafePork project site or has an established working relationship with the International Livestock Research Institute project staff.

Using a purposive sampling approach, the key informants in Hanoi were identified through personal networks of colleagues working with the SafePork project in Vietnam. To recruit participants in Thai Nguyen Province, an active snowballing approach was used with key informants identified through personal networks of colleagues with provincial governments and universities in human and animal health sectors. Actors contacted were asked to provide details of persons involved in AMR projects.

The number of interviews was determined to ensure representation across human health and animal health sectors and by the concept of saturation, that is, the number of participants considered adequate to represent the situation and to reach the point that no new information was being obtained from interviews [27].

In total we approached eight actors from Thai Nguyen Province and 18 from Hanoi who had been identified to be working across the animal and human health sectors. Three people from Thai Nguyen and six in Hanoi chose not to participate in this study. Overall, 17 interviews were conducted with 12 informants in Hanoi and five in Thai Nguyen province. One of the interviews in Hanoi included two female participants from the animal health sector and one of the interviews in Thai Nguyen included two male participants from the animal health sector. Information about the purpose of the study and that they could withdraw or cease the interview at any time was given to interviewees prior to the interview. Written consent was obtained by the interviewees before the interview was conducted. All participants gave their permission for the interviews to be recorded and the interviewer guaranteed the privacy of participants.

### Interview guide

Two semi-structured interview guides, one for animal health sector and one for the human health sector, were developed in English, translated into Vietnamese and pilot tested in Hanoi. Interviewees were chosen on the basis of availability and included: one Vietnamese and one non-Vietnamese professional working respectively in the animal health and human health sectors. The interview guides were subsequently refined before commencement of study interviews (additional file 1). The guides comprised entirely of open-ended questions and had four sections: 1) participant and organisation involvement in AMR projects; 2) investigation process; 3) resources available; and 4) inter-sectoral collaboration.

Sections one and four of the interview guides were the same for both sectors and differentiated only with sections two and three. The interview guides included three questions in section one and three questions in section two. Section three included six questions in the human health sector guide and nine in the animal health sector guide. Both the animal and human health sector participants were asked eight questions in section four. Two hypothetical situations were developed to guide the semi-structured interviews during sections two and three of the interview guides for both sectors. The hypothetical scenarios were designed to provide participants from different levels of government, non-government and professional background a scenario based on a common issue [28]. Participants working within the human health sector were given the hypothetical scenario of the “Identification of resistant pathogen in a person during a foodborne illness outbreak”. For participants working within the animal health sector, they were given the hypothetical scenario concerning the “Identification of resistant pathogen within the pork-value chain, including: slaughterhouse, retailer and pork product”. The semi-structured interviews were conducted by MM and for interviews conducted in Vietnamese a translator provided translation from Vietnamese to English. Written notes during the interviews were taken in English and/or Vietnamese and interview length ranged between 30 to 90 min. All interviews were recorded, transcribed and translated to English if needed by an external company professional transcriber or one of the authors. Four translators were briefed on the research project prior to the interviews. All translators had previous experience working across One Health and AMR research in Vietnam. To attain validity of the research interpretations, peer debriefing and critique of fieldwork notes was conducted through meetings and discussions of informant’s perspectives with the research team post interviews.

### Data management and analysis

Analysis of qualitative data was performed using Microsoft Office, Word Processing and Excel Office 16 using the guide by Braun and Clarke, 2006 [29]. Qualitative thematic analysis was performed by the primary author of this study. To accurately report the situation and the results, MM was based in Vietnam and spent substantial effort to immerse herself within the data and subject matter. The interview transcripts from all interviews were read-and re-read to familiarise the author with the data. The dominant ideas from four interviews from Hanoi and one interview from Thai Nguyen province were identified by detecting the consistent patterns in the data. The central ideas that captured the participants perspectives were reviewed and then applied to the remaining interviews. The common points of difference and of similarity in the data were identified and built into codes. The identified codes were developed and labelled to systematically sort the meanings of the text and the relationships between the participants narratives. The codes were entered into a codebook with full definitions and code application directions, and modified as new information and insights were gained [30]. The codes that captured the prominent ideas throughout the data were built into themes. The coding and theme development were revisited and refined consistently throughout the process [31]. The coding was led by author MM with continuous communication and cross checking with TL and adjustments were made when appropriate after discussion with all authors.

## Results

In this study, a total of 17 participants in 15 semi-structured interviews were conducted. Eleven semi-structured interviews with 12 informants were conducted in Hanoi and four interviews with five participants were conducted in Thai Nguyen province. Participants in Hanoi included eight (six women and two men) from the animal health sector and four (three men and one woman) from the human health sector. In Thai Nguyen, the interviews included one woman and two men from the animal health sector and two men from the human health sector (Table 1 and Table 2).

**Table 1:** Number of participants by agency for the 17 participants in interviews conducted in 2018–2019 in Vietnam

Agency	Human health	Animal health
Government research agency	1	3
Ministry of Health / Ministry of Agriculture and Rural Development	1	3
Sub-Department within ministry	1	2
University	1	1
Hospital	1	–
International organisation	1	2
TOTAL	6	11

**Table 2:** Demographic characteristics of the 17 participants in interviews conducted in 2018–2019 in Vietnam

Demographic	N (%)
Gender	
Male	12 (70.6%)
Female	5 (29.4%)
TOTAL	17
Hanoi province	12 (70.6%)
Thai Nguyen province	5 (29.4%)
TOTAL	17
Animal health sector	11 (64.7%)
Human health sector	6 (35.3)
TOTAL	17

**Fig. 1:**
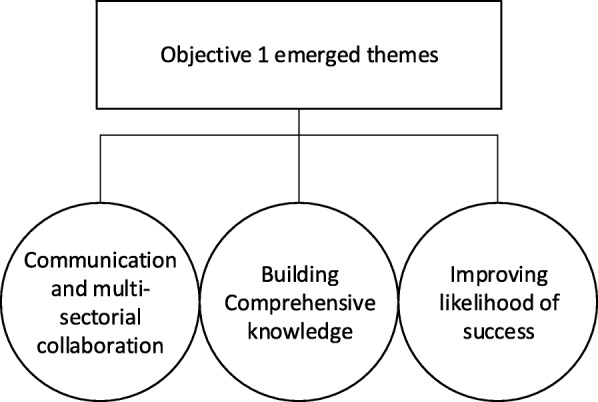
Themes emerged of the potential advantages of cross-sectorial collaborations between key informants from the animal and human health sectors in Vietnam

**Fig. 2:**
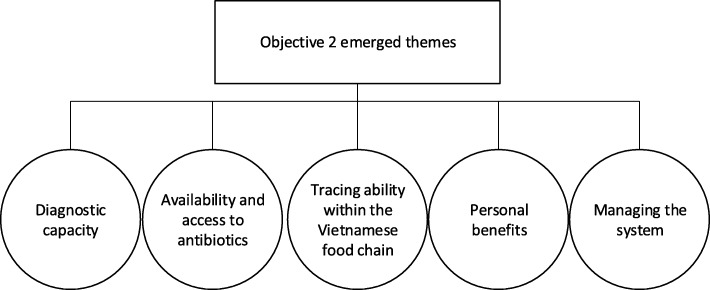
Themes emerged of the potential challenges that key informants in the animal and human health sectors face during an investigation of AMR within the pork-value chain in Vietnam

From the analyses of interview transcripts, eight themes have emerged (Fig. 1; Fig. 2; Table 3) Three themes were built from participants perspectives on a One Health approach and cross-sectoral collaboration; (1) Communication and multi-sectorial collaboration; (2) Building comprehensive knowledge; (3) Improving likelihood of success. Five themes emerged from participants views of the challenges to investigate antibiotic resistance: (4) Diagnostic capacity; (5) Availability and access to antibiotics (6) Tracing ability within the Vietnamese food chain; (7) Personal benefits and (8) Managing the system.

**Table 3:** Themes from participant perspectives of a One Health approach and challenges to investigate AMR in Vietnam from the 17 interviews conducted in Hanoi and Thai Nguyen Province during 2018–2019

Theme	Example
Communication and multi-sectorial collaboration	*“The gap in the connection…that’s why right now it’s hard for us to do it. Because we lack the resource to link the people…the division together”* Man, animal health sector
Building comprehensive knowledge	*“Have the connections, between human and animal…enhancing the capability in researching, in knowledge... the antibiotic resistance between human and animal... overall...then we may... further... research, there would be the learning from each other”.* Woman, animal health sector
Improving likelihood of success	*“this man investigates into this and that man investigates into that, but you must have the exchange of information between each others... yet now... there is no sharing, no collaboration for the next step, so it would properly be not effective”,* Woman, animal health sector
Diagnostic capacity	*“there are multiple difficulties, but especially the problem of gene analysis, genotype, Vietnam can implement some, but some has not, because of no machinery, no solvent, no chemicals; then it must be transferred overseas for analysis”* Man, human health sector, *“there need’s more well-trained professionals… in antibiotic resistance… [in] deep analysis is also insufficient.”* Woman, animal health sector
Availability and access to antibiotics	*“we have the national regulation and we apply to the public... but it (does) not work yet”*, Man, animal health sector
Tracing ability within the Vietnamese food chain	*“the traceability of a potential source is difficult.... 90% would be bought at wet market, where related organizations such as Department of Trade cannot control”.* Man, human health sector
Personal benefits	*“The farmers even concern more about their economic, they are willing to use AB to prevent disease. In livestock law, in purpose of preventing disease, young animal can use AB, but the definition for “young” by month of age is not clear. Some regulations on how long farmers should stop using AB before slaughtering but farmers do not strictly abide”.* Woman, animal health sector
Managing the system	*“You know sometimes, Vietnamese people…. It really works if you have personal relationship or if you have good relationship before”.* Man, human health sector

Results highlight that there is moderate variation of perspectives between the provincial and national governments on what is needed to achieve a One Health approach to a surveillance system and to improve collaboration and communication across sectors. At the provincial level, the participants believed that for a One Health approach to be implemented, higher government and management support was essential. This could be in the form of management support for employees to meet across sectors and build relationships. The view of participants working at the national government level differed where support for a One Health approach was primarily in relation to financial resources.

Many participants spoke of the difficulty of addressing AMR due to: limited resources and capacity to investigate AMR, misuse and abuse of antibiotics in the human and animal health sector, poor traceability of pork products in the food system, livelihoods dependent on benefits from antibiotic sales and consumption, and a convoluted governance system.

### One Health approach

#### Communication and multi-sectorial collaboration

This theme evolved from the varied opinions about the level of information sharing and collaboration between the animal and human health sectors.

A participant working within an international organisation spoke of the limited collaboration between the sectors, he mentioned, *“we [the organisation] felt there was a lack of intersectoral communication regarding AMR surveillance”.* (Participant 1, man, human health sector). It was recognised by one senior official that, *“the most difficult thing lays in the intersectoral collaboration, at the same moment, focus on the investigation together to give out the result, that is the difficulty”* (Participant 5, man, human health sector). One animal health government researcher described the lack of respect the animal sector received, and it was this that caused barriers between collaborations; *“the Veterinary Health department, it has not been called…taken care of seriously, therefore for example, many times when I go for connections [seek collaboration], they often don’t want to meet us, and they are not willing to share information also, or to collaborate.”* (Participant 6, woman, animal health sector). Many participants spoke of personal relationships between themselves and actors working in another sector. These informal relationships and networks were key support networks in knowledge sharing, processing requests, gaining new knowledge and learnings. One participant felt that he worked with a One Health approach as he had relationships with other members working across sectors. However, relationship building across sectors may or may not be supported by their higher management or employer. At the provincial level, one participant spoke of the challenges in developing relationships with the other sector because their manager did not feel it was a necessary endeavour.

#### Building comprehensive knowledge

This theme emerged from participants views of the shared benefits in cross collaboration. The benefits gained included sourcing new information, understanding AMR from a different perspective and having a deeper awareness of the interconnecting and interdependent factors. Twelve of the 17 participants referred to a One Health approach to enabling *“a fuller understanding”* or a *“more complete view”* of the AMR situation in Vietnam.

One participant spoke of absence of team work in addressing AMR amongst the government sections. Increasing the cross-sectoral training was thought to be a mechanism to increase team work and collaboration and to ultimately build knowledge across the sectors, as one participant from the provincial level mentioned, *“we can see different perspectives from different sectors for the overview of current [AMR] situation”* (Participant 16, woman, animal health sector).

From the participants perspectives, sharing of information was beneficial; however, some participants also expressed the need for increased resources to enable collaboration and increase sector knowledge. *“It’s the biggest issue, because in our country we have very limitation on the resources so when we work in the One Health [framework] we can share data, share information and publication our work”* (Participant 11, woman, human health sector).

#### Improving likelihood of success

This theme evolved from the participants perspectives on how collaborative efforts between the human and animal health sectors would progress each sectors’ AMR strategies and objectives. There was slight variation between members of international organisations and Vietnamese government organisations regarding the level of multi-sectorial collaboration needed. However, participants did relay that sharing information and having an understanding of each sectors activities does enhance their capabilities to achieve their goals.

Sharing information as part of the One Health approach is one of the first steps to begin a One Health approach. The perspectives of participants working at the international level spoke of the requirement for both sectors to have an understanding of the actions taken by the other sector. One Participant noted *“it is important to show each other that everyone is doing efforts in each sector, in its domain and to stop the name shaming and blaming the animal health sector”* (Participant 3, woman, animal health sector).

At the national and provincial government level, perspectives focused on when collaboration was required and limited level of cohesion between investigative methods and analysis. Five of the participants spoke of the need for a One Health approach to be established at every level of an AMR surveillance system to coordinate surveillance protocols, data collection and analysis and share information across sectors. For example, one national government employee from the human health sector spoke of *“all must have the intersectoral collaboration. For instance, the problem in the stage of, that is, to evaluate the current situation also needs the intersectoral collaboration. Secondly, in the stage of develop the cooperative solution, there also need the intersectoral collaboration. And after that, it also need [s] the measurement on the impact, the result, and the cooperation of the, the sectors”* (Participant 5, man, human health sector).

### Antibiotic resistance within the pork value-chain

#### Diagnostic capacity

There were differing opinions on the diagnostic capacity of the animal health and human health sectors; including, the human resources available, equipment and laboratory capacity and the available finances. At the provincial level, the participants spoke of the need to send samples to Hanoi for further testing, time constraints and the high financial cost. From a national perspective, participants stated instances of not having the appropriate equipment, adequate human resources or finance to conduct some laboratory tests and that samples are then sent for testing overseas. Within the hospital system, the participant spoke of the limited ability to isolate patients and inadequate financial resources causing difficulty and delays to conduct the most appropriate tests, “*the condition in our country Vietnam it, the infrastructure it is not as good as the overseas. Don’t know that… there is no ward for isolation, no sanitary ward, that’s it.”* (Participant 12, man, human health sector) There were slight variations in the opinions by participants working at the international level; however, it was acknowledged that there are challenges for laboratory workers.

One participant felt *“microbiology is not used as much in Vietnam as it would be in European countries. Not everybody who comes to the hospital with a suspicion of infectious disease is cultured [have samples cultured]. You wouldn’t find everything”.* (Participant 1, man, human health sector)*.*

Participants from the animal health and human health sectors spoke of the challenges in acquiring sufficient supplies and resources to perform laboratory tests. The challenges included the large amount of paperwork, limited supplies and poor equipment. However, in contrast one officer in animal health management noted that it is easy for people in the field to collect the samples and conduct the most appropriate test. She believed that this was similar to the situation when collecting samples for antibiotic residue testing, “*[here] we do the coordination… we design then we ask them to do this and that, so they proceed”.* (Participant 8, woman, animal health sector).

#### Availability and access to antibiotics

This theme emerged from the participants view of the ease of access to antibiotics by the general public. Participants from both sectors spoke of the lack of enforcement of antibiotic regulations, resulting in the abuse or misuse of antibiotics. This was seen by participants as a fundamental driver of antibiotic resistance. Some participants spoke of the need to increase awareness of AMR to the general public, the challenges of substandard antibiotics in the market, and the lack of private sector and government accountability.

A participant at the national level spoke of how in Vietnam you can *“still buy antibiotic without prescription…If you want. Don’t need doctor… it’s quite [a] challenge if, we don’t have policy… we don’t have legal regulation framework for antimicrobial resistance. It’s still [a] challenge. And it should be, belong to our government not from the international”.* (Participant 11, woman, human health sector).

#### Tracing ability within the Vietnamese food system

This theme evolved from the participants ideas on the challenges that an investigation in antibiotic resistance poses within the context of the pork value-chain.

One worker from the animal health worker in Hanoi stated, *“tracing back origin, presently… it seems, in the surveillance program of ours, we can trace it back, but in reality, it is difficult”.* (Participant 4, man, animal health sector).

There were varied opinions between the sectors on which stages of the pork value-chain it would be easier to trace the pork meat. From the participants view, it can be difficult to identify the trajectory of pork meat through the value chain. A national government employee remarked that if the sample is taken at the slaughterhouse, there is a chance to trace it back to the farm. However, if the sample is taken at the wet (traditional) food market, traceability is difficult. One participant from the provincial level mentioned that, *“If the sample comes from a farm or super market, it is easy to know the origin, but if it is from wet market, it is just [not] possible” (*Participant 16, woman, animal health sector).

#### Personal benefits

The theme of personal benefits evolved from participant perspectives, that a central cause of antibiotic resistance was the misuse of antibiotics on farm. Participants relayed that farmers seek their own financial benefit by use of antibiotics to reduce pig losses and maximise growth rather than considering reducing antibiotic resistance for the longer-term benefit of animal health and human health. Immediate financial benefit for those selling antibiotics was also recognised.

One officer in the animal health sector reported, *“they are selling antibiotic, is also a big income for the animal health worker or the veterinarian, so maybe they decide to give the antibiotic, even multiple antibiotic… so they can sell to report the case, or to do the investigation and conduct the sensitivity test before they are coming up with a good what we call prescription”.* (Participant 2, woman, animal health sector).

Justification of the pig farmers actions was thought to be that the pig farmers may not have information available or have adequate understanding of appropriate dosage amounts and of withdrawal times. One participant in the animal health sector mentioned.

*“Because… I go down to the farms, I saw that there are many people they, it means that in the feeding brand there has the antibiotics, then the antibiotic powder, they also unintentionally…”* (Participant 4, man, animal health sector).

#### Managing the system

This theme grew from the participants insights into the benefits from their personal relationships and networks and their ability or inability to access information or progress an investigation.

Although it seemed the sectors were siloed, and participants felt there were barriers to connecting with employees from the other sector, this was overcome through their own personal relationships. At the provincial level, the close connections [with the local authority] enable investigation of AMR to go *“more smoothly”.*

One participant from the national level spoke of the challenges in connecting with the local province and working with officials with whom they had no relationship. Although documents or requests would ultimately be addressed, it would take a significant longer period of time if there was no established relationship. The participant relayed that when *“we can carry on at the local areas or the agencies… That collaboration… takes quite a time, tough… to go well in the locality in Vietnam, following the custom of Vietnam, there must be the acquaintance and … good relationship”.* (Participant 7, woman, animal health sector).

## Discussion

The aim of this qualitative research study was to explore and elucidate key informant perspectives on the potential benefits of a One Health approach to a surveillance system and to identify potential challenges of investigating AMR in Vietnam using the pork value chain as a case study. The themes presented illustrate the complexity of multi-sectorial collaboration, and the multiple factors that underlie the challenges of investigating AMR along the pork value chain in Vietnam.

### One Health approach

Antibiotic resistance has been deemed a quintessential One Health problem [17]. Based on the perspectives from participants, there is a desire for a collaborative strategy to address AMR to be realised; however, there are existing challenges in the implementation. These findings are consistent with studies elsewhere that have highlighted the challenges States face in implementing their National Action Plans on antimicrobial resistance. Challenges associated with performing One Health surveillance may persist from the design stage, to execution and through to monitoring and evaluation due to the poor availability of resources and personnel [32].

Poor data quality and insufficient comparable data on AMR in the human and animal health sectors are a key barrier to accurate comparisons between human and livestock populations and between countries and regions [26]. Participants believed that once these are overcome, information sharing across sectors may increase. The challenges of collaboration and information sharing between sectors have been reported elsewhere in Asia, where cooperation may be written in policies but lacking in practice [33]. Information sharing amongst sectors in this study was highly dependent on informal relationships rather than the collaborations based on formal policies. The participants at the provincial level spoke of the limited financial support to undertake interdisciplinary work or collaborate with other sectors due to institutional barriers set by higher management. Limited resources allocated to facilitating cross-sectorial collaboration has been a reported barrier to enabling networks [34]; although, it is essential to support the establishment of networks amongst the different levels of government, non-government and international organisations.

Government agencies have pledged their commitment through the development of two National Action Plans (one in the animal and one in the human health sector) and convened a National Steering Committee on Antimicrobial Resistance involving actors from across the human and animal health sectors [21, 22]. Yet many of the participants believed there was limited commitment by the Vietnamese government to address AMR. From the perspective of the participants in this study, this lack of uniformity in the government’s National Action Plans on antimicrobial resistance and the limited number of meetings between the steering committee signalled a lack of commitment by the government. Without a more unified approach between sectors, it may create difficulties for partnerships and information sharing to develop lower down the hierarchy. This was highlighted by participants working at the provincial level, who continuously mentioned that they need direction and confirmation from National senior management if they are to collaborate with other sectors. However, one participant mentioned involvement in the One Health activities in Thai Nguyen as part of the Vietnam One Health University Network and stated that this created valuable insight and networking opportunities which would not have been possible otherwise. Academic institution level strategies that develop skills in interdisciplinary training may be able to be translated to government agencies. Participatory modelling approaches to improve cross-sectorial collaborations have been advocated previously for South East Asia, with provincial governments being a key focus area [35].

In this study, the majority of the participants reported that the One Health surveillance system should be developed with all sectors involved from the design stage. The participants perceived a One Heath approach to strengthen their capacity to achieve their own sectors objectives to tackle AMR by gaining new knowledge and understanding. To achieve successful multi-sectorial participation, the development of trust, transparency, equal representation and consensus amongst all relevant sectors is needed [24]. Bordier and Nguyen (2017) reported that actors working in the animal and human health sectors have found it difficult to understand each sectors’ surveillance objectives, leading to a lack of mutual understanding and of the shared benefits of consistent collaboration [25]. Evaluation frameworks which encompass these interdisciplinary outcomes can help capture and highlight the benefits of multi-sectorial collaboration [36].

### Antibiotic use and antibiotic resistance in the pork-value-chain

Although antibiotic use was not a focus of this study, several of the respondents perceived that the antibiotic use within food production was the key driver of antibiotic resistance within humans. One participant from the animal health sector stated *“…the use of antibiotics in the animal… leads to the resistance… we know that in Vietnam, a lot of antibiotic… used in husbandry”* (participant 10, man, animal health sector). Secondly, one interviewee from the health sector proposed that the level of resistance witnessed within the human healthcare sector is emerging from antibiotic use in animal production.

Antimicrobial resistance is a growing threat to human and animal health with a disproportionate burden in low-to-middle-income countries (LMIC) [37]. Often LMIC’s have a high burden of infectious diseases, poverty and weak governance and health systems [37]. These prevailing conditions are aggravated by low awareness about antibiotics and its resistance, poor access to health services, easy availability of antibiotics over the counter, pleural health care with delayed, inappropriate and sub-standard treatments [37, 38]. Inappropriate use of antibiotics in LMIC have been linked to the lack of diagnostics and poor infrastructure; industry incentives and advertising; and the economic benefits for the prescriber [39–42]. In this study, participants mentioned the difficulties in diagnostic capabilities, the ready accessibility of antibiotics without a prescription and the prescription and use of antibiotics for financial gain. The limited resources to undertake diagnostics in hospitals can lead to healthcare professionals prescribing antibiotics before an infection is diagnosed [43]. Healthcare professionals have been reported to prescribe and dispense antimicrobials to improve patient wellbeing due to insufficient infrastructure and poor hygiene in the health system [5, 39].

In comparison to many high-income countries (HIC), access to antibiotics is highly unregulated in LMIC, were consumers can purchase antibiotic treatment from authorised and unauthorised pharmacies without a prescription [7, 43]. Evidence has shown that the use and misuse of antibiotics may be associated with broader sociocultural [44] and sociodemographic factors [45]. In the Philippines, a study by Barber et al., found accessing unprescribed antibiotics from the local *sari sari (small retail business)* stand and antibiotic sharing amongst family and friends was a common practice [46]. Antibiotic prescribers are also found to be influenced by their patient’s sociodemographic factors, and may adapt treatment to patient’s income, antibiotic accessibility in the area and their patient’s medication history [43].

High, poorly regulated human access to antibiotics raises particular concern about the contribution of human health to AMR, and about antibiotics manufactured for use in human health being given to animals. Less control over antibiotic use in both the animal health and the human health sectors means that respective contribution to AMR is not known, and usage in the animal sector may or may not be more of a contributor to AMR development. As part of the National Action Plan, to monitor antibiotic use within the animal health sector, the Department of Animal Health will generate monthly reports of the amounts of antibiotics imported and sold in Vietnam by pharmaceutical companies [22].

The international community has placed emphasis on the curtailment of inappropriate use of antibiotics within the human health sector and elevated antibiotic resistance to its current status as a global priority [47]. Despite the growing concern between the link of antibiotic resistance and antibiotic use within the animal food production sector, this discourse only began recently with the continuation of human health as the central context of AMR containment strategies [17, 48]. As AMR increasingly becomes defined as a security threat to human health, this may result in human health being the dominant concern in efforts to curb the spread of resistance pathogens [33]. Knowledge sharing protocols and methods across sectors and establishing routine collaboration can help promote collaboration between relevant stakeholders and across silos [35, 49].

The participants in this study highlighted several challenges to investigate antibiotic resistance along the pork value-chain in Vietnam, including the difficulties of tracing food in the informal food system and laboratory capacity to conduct testing. They spoke of the complexity of tracing pigs and pork products within the food system in Vietnam and the difficulty in determining the movement of pigs and of pork products from farm of origin to the consumer. One interviewee suggested that there are not sufficient records kept which impedes investigations. Animal and animal product traceability in high-income country contexts, such as Australia, are underpinned by systems for farm registration, animal identification (at individual or batch level) and animal movement records that at present are insufficient in Vietnam.

The Vietnamese government is currently working in partnership with international collaborators and regional networks to establish the national reference laboratories for AMR surveillance. The Ministry of Agriculture and Rural Development acknowledge that the management of regulations of antibiotic use in the food production sector has been inadequate [22]. The participants in this study had different perspectives on the capacity of the laboratories working on AMR. Most participants working at the managerial position believed the capacity of laboratories was adequate to undertake the required tests needed. Interviewees working in the laboratory held a different perspective and acknowledged having to send samples overseas for analysis or having to adjust methods due to the condition of equipment. The interviewees at the provincial level did not have any current AMR projects. In their view, they had adequate equipment for testing; nevertheless, they also spoke of previously sending samples to Hanoi for further testing. On the positive, participants mentioned that they do have access to information within their organisations and can communicate with other laboratories. Although not specifically mentioned in this study, collaborative networks between laboratories, particularly the use of laboratory information systems (LIS) is recognised to improve capacity and streamline data systems for AMR surveillance [50].

## Limitations

There are several limitations to this study. Firstly, the study focuses on a One Health approach; however, we do not include the environment sector. This was due to the limited activity addressing antibiotic resistance in Vietnam by the Ministry of Natural Resources and Environment and thus the authors felt that it would be difficult to gain information on antibiotic resistance.

Secondly, during the course of this research study, the hypothetic scenarios used as case studies for the interviews may not have been realistic or a plausible scenario at the time. The antibiotic resistance surveillance system in Vietnam is still in its infancy and therefore many of the participants perspectives were based on what would hypothetically happen.

Thirdly, at the provincial level there were no reported activities or investigations into antibiotic resistance in Thai Nguyen province. The participants opinions may have related to their experiences of working on specific research projects or their current work in addressing antibiotic residues within the pork value-chain.

## Conclusion

The aim of this project was to elucidate information relating to the benefits of a One Health approach to address AMR in Vietnam, and the challenges during an investigation within the context of resistant foodborne bacteria found within the pork value chain.

The themes that evolved from participants views highlight that across the human health and animal health sectors, actors face similar challenges to investigate AMR and see the benefits a One Health approach offers. The challenges to the investigation of AMR that participants raised in this study included the lack of traceability in the pork value chain, and the limited regulation of antibiotics in the human and animal health sectors. The different views on diagnostic capacity between sectors and levels suggests that a deeper understanding of the challenges they face may assist in the development of the AMR surveillance system. Although both sectors spoke of limitations in ability to perform the most appropriate tests, the results indicate the need to strengthen the animal health sector is more pronounced.

This study demonstrated that there is potential to strengthen multi-sectorial collaboration between the animal health and human health sector in Vietnam. The perceived benefits of a One Health approach were information sharing and collaboration, which were seen to increase respective sector ability to achieve their objectives related to tackling AMR. Current focus is to strengthen the relationships at the top level of government. However, we recommend an inclusive approach to multi-sectorial communication supported by government network activities to facilitate partnerships and create cross-disciplinary awareness and participation that can build on promising existing informal networks and collaboration.

## Supplementary information

**Additional file 1.** Interview guide

## Data Availability

The datasets used and/or analysed during the current study are available from the corresponding author on reasonable request.

## Abbreviations

AMR: Antimicrobial resistance
HIC: High-Income Country
LMIC: Low-to-Middle-Income Country
MARD: Ministry of Agriculture and Rural Development
WHO: World Health Organization
