# Supplementary material for: The challenges of investigating antimicrobial resistance in Vietnam - what benefits does a One Health approach offer the animal and human health sectors?
Source: BMC Public Health. 2020 Feb 11;20:213. doi: 10.1186/s12889-020-8319-3 (PMC7014660; doi:10.1186/s12889-020-8319-3)
Supplement: Supplementary file 1 — Additional file 1. Interview guide [file 12889_2020_8319_MOESM1_ESM.docx]

| **Interview outline for scenario 1 Hypothetical event:** Identification of resistant pathogen found in a person during a foodborne disease outbreak in a hospital. | |
| --- | --- |
| Part 1. Background | |
| 1. Is your organisation currently involved in addressing antibiotic resistance? If yes, how? |  |
| 1. How many people are involved in investigating antibiotic resistance in your organisation? |  |
| 1. What are the steps involved in assessing for the risk of an antibiotic resistant pathogen in Viet Nam? |  |
| Part 2. Investigation Process | |
| 1. 1. Based on the scenario, what investigation would occur to identify the potential source/s of exposure to the resistant pathogen? | |
| 1. Describe the steps in the investigation. | |
| 1. Would it be easy to conduct this investigation? 2. If Yes – Why? 3. If No – Why? | |
| Part 3. Resources Available | |
| 1. What types of resources are needed for this investigation? | |
| 1. What is the capacity in the agency to;    1. Access relevant data?    2. Communicate with laboratory and other agencies? | |
| 1. Are there any limitations involved in performing the most appropriate test? If so, can you give an example? | |
| 1. Does the person in role of investigating antibiotic resistant pathogens currently have the resources to collect samples? | |
| 1. How are the samples collected?    1. Based on the level of risk    2. Based on budget | |
| 1. Has this type of event occurred before? If Yes - What happened at that time? | |
| Part 4. Intersectoral Collaboration | |
| 1. Have you heard about the One Health concept? If yes, do you have an example of a One Health activity in your agency? | |
| 1. If yes, what are the benefits of the One Health approach? | |
| 1. What value does the One Health approach to surveillance of ABR provide to your agency? | |
| 1. From your perspective, at what stage of the surveillance system, do you require intersectoral collaboration? | |
| 1. In your opinion, what doesn’t the One Health Approach to an ABR Surveillance System consider? | |
| 1. Are there any barriers in implementing intersectoral ABR surveillance activities? If so, can you give an example? | |
| 1. Having these limitations, what possible interventions do you need to establish a OH Surveillance System in Viet Nam? | |
| 1. What resources would be useful to improve integrated ABR surveillance? | |

| **Interview outline scenario hypothetical event:** Identification of resistant pathogen in a pig product within the pork-value chain? |
| --- |
| Part 1. Background |
| 1. Is your organisation currently involved in addressing antibiotic resistance? If yes, how? |
| 1. How many people are involved in investigating antibiotic resistance in your organization? |
| 1. What are the steps involved in assessing for the risk of an antibiotic resistant pathogen in Vietnam? |
| Part 2. Investigation Process |
| 1. Based on the scenario, what investigation would occur to identify the potential source/s of exposure to the resistant pathogen? |
| 1. Describe the steps in the investigation. |
| 1. Would it be easy to conduct this investigation?    1. a. If Yes – Why?    2. If No – Why? |
| Part 3. Resources Available |
| 1. What types of resources are needed for this investigation? |
| 1. What is the capacity in the agency to;    1. Access relevant data?    2. Communicate with laboratory and other agencies? |
| 1. Are there any limitations involved in performing the most appropriate test? If so, can you give an example? |
| 1. Does the person in role of investigating antibiotic resistant pathogens currently have the resources to collect samples? |
| 1. How are samples collected?    1. Based on the level of risk    2. Based on the budget available |
| 1. Has this type of event occurred?    1. If Yes - What happened at that time? |
| 1. At this time – how could this event occur? |
| 1. What, if any, testing of pig product is being done that may identify RP on pig product? |
| 1. How does your organisation share relevant information with other sectors involved in an ABR investigation? |
| Part 4. Intersectoral Collaboration |
| 1. Have you heard of the One Health concept? If yes, do you have an example of a One Health activity in your agency? |
| 1. If yes, what are the benefits of the One Health approach? |
| 1. What value does the One Health approach to surveillance of ABR provide to your agency? |
| 1. From your perspective, at what stage of the surveillance system, do you require intersectoral collaboration? |
| 1. In your opinion, what doesn’t the One Health Approach to an ABR Surveillance System consider? |
| 1. Are there any barriers in implementing intersectoral ABR surveillance activities? If so, can you give an example? |
| 1. Having these limitations, what possible interventions do you need to establish a OH Surveillance System in Viet Nam? |
| 1. What resources would be useful to improve integrated ABR surveillance? |
